# Association of Basal Metabolic Rate and Nutrients Oxidation with Cardiometabolic Risk Factors and Insulin Sensitivity in Sedentary Middle-Aged Adults

**DOI:** 10.3390/nu12041186

**Published:** 2020-04-23

**Authors:** Francisco J. Amaro-Gahete, Lucas Jurado-Fasoli, Jonatan R. Ruiz, Manuel J. Castillo

**Affiliations:** 1 Department of Medical Physiology, School of Medicine, University of Granada, 18071 Granada, Spain; juradofasoli@ugr.es (L.J.-F.); mcgarzon@ugr.es (M.J.C.); 2 PROmoting FITness and Health through Physical Activity Research Group (PROFITH), Department of Physical Education and Sports, Faculty of Sport Sciences, University of Granada, 18071 Granada, Spain; ruizj@ugr.es

**Keywords:** metabolic rate, basal metabolism, fat oxidation, carbohydrate oxidation, indirect calorimetry, cardiometabolic risk, energy balance, insulin resistance

## Abstract

This cross-sectional study aimed to examine the association of basal metabolic rate (BMR) and basal fat and carbohydrate oxidation (BFox and BCHox, respectively) with cardiometabolic risk factors and insulin sensitivity in sedentary middle-aged adults. A total of 71 healthy sedentary adults (37 women) aged 40–65 years participated in the current study. Data were collected during the baseline assessments of the FIT-AGEING randomized controlled trial. BMR was measured via indirect calorimetry, and BFox and BCHox estimated by stoichiometric equations. Blood pressure, glucose, insulin, total cholesterol, high-density lipoprotein cholesterol, low-density lipoprotein cholesterol, and triglycerides plasma levels were selected as cardiometabolic risk factors and assessed following standard procedures. We observed positive associations of BMR with plasma insulin and the homeostatic model assessment of insulin resistance index (HOMA; all *p* < 0.05) which were attenuated or disappeared after controlling by sex, age, and/or lean mass. There were positive associations between BFox and the quantitative insulin sensitivity check index (QUICKI; *p* < 0.015), while negative associations were noted between BFox and plasma insulin and HOMA (*p* < 0.015). There was a significant negative association between BCHox with QUICKI (*p* < 0.01), whereas significant positive relationships were obtained when BCHox was associated with plasma insulin and HOMA (*p* < 0.01). These associations persisted in almost all cases when controlling by sex, age and/or lean mass. No further relationships were found when BMR, BFox, and BCHox were associated with other cardiometabolic risk factors. In conclusion, our study findings support that greater BFox and lower BCHox are related to improved insulin sensitivity, whereas BMR seems to be not associated with neither cardiometabolic risk nor insulin sensitivity in sedentary middle-aged adults. Further intervention studies are necessary to well-understand the physiological mechanism implied in this relationship.

## 1. Introduction

The important increment of life expectancy during the last decades in developed countries has triggered a subsequent rise of cardiometabolic risk and cardiovascular diseases’ incidence [1], which is currently the most significant cause of mortality [2]. Patients with chronic cardiometabolic diseases (e.g., metabolic syndrome, obesity, or type 2 diabetes mellitus) and cancer present several energy metabolism impairments suffering metabolic inflexibility, which is understood as a deficient capacity to adapt energy availability to environmental demands [3].

Basal metabolic rate (BMR) accounts for 60–70% of total energy expenditure [4,5], and it decreases progressively with increasing age [6]. Aging is also characterized by changes in body composition, mainly a reduction in muscle mass, which is the most important contributor to the progressive decrement of BMR [6]. An increased BMR is associated with improvements in body composition and cardiometabolic risk factors [7], thus strategies to increase BMR should be taken into account during the aging process [8].

In fasting conditions, the decrement of dietary carbohydrates and fats in combination with a decline of insulin glucagon ratio promotes a switch toward fatty acid oxidation [9]. White adipose tissue is the predominant source of fatty acids in response to fast that are used in the periphery as fuel [9]. In this sense, the ability to store fatty acids during caloric availability and to release fatty acids during caloric restriction has been proposed as a key factor of metabolic flexibility [3]. Recent studies have suggested that an increased fat oxidation and a decreased carbohydrate oxidation in fasting conditions (i.e., basal fat oxidation (BFox) and basal carbohydrate oxidation (BCHox), respectively) are associated with a lower risk of cardiometabolic disease in patients [10], yet whether this applies to adults free of disease is currently unknown.

Previous studies have shown a positive association of BMR with blood pressure [11,12,13], triglycerides [14], glucose [15,16], and waist circumference [17]. Low BMR is a predictor of the development of obesity and its metabolic complications including cardiometabolic diseases [18,19,20]. However, subjects with an energy sparing condition could present cardiometabolic risk [19], inducing metabolic adaptation through the decrease in thermogenesis (i.e., BMR) [21]. Total daily energy expenditure has an impact on body weight regulation and general health, specifically on metabolic health [7]. BMR accounts for about 60%–70% of total daily energy expenditure and is responsible for overall energy homeostasis [22]. About 60% of the BMR is explained by lean mass, being the main tissue that possesses the mitochondrial capacity for energy expenditure [22]. Higher lean mass, and consequently higher BMR, produces the utilization of glucose or lipid to produce energy [22]. This high energy metabolism could exert regulatory actions in different tissues (e.g., liver, muscle, and adipose tissue) acting as dynamic and active tissues regulating several cardiometabolic parameters and achieving a low cardiometabolic risk [23].

Metabolic inflexibility is at the core of the pathophysiology of metabolic diseases, due to the deterioration of the insulin-mediated substrate switching [10]. However, there are scarce studies that investigate the association of energy metabolism parameters related to metabolic flexibility in fasting (i.e., BMR, BFox, and BCHox) with cardiometabolic risk factors and insulin sensitivity in middle-aged individuals still free of chronic diseases. Therefore, this study aimed to examine the association of BMR, BFox, and BCHox with cardiometabolic risk factors and insulin sensitivity in sedentary middle-aged adults.

## 2. Materials and Methods

### 2.1. Design and Participants

This cross-sectional study was performed under the framework of the FIT-AGEING randomized controlled trial [24]. Advertisements in electronic media, social networks, and leaflets were used to recruit the participants of the study. Exclusion criteria were: being physically active (more than 20 min of moderate–vigorous physical activity on more than 3 d/week) [25], being participants of a structured exercise intervention during the last 3 months, presenting a non-stable body weight (i.e., >3 kg) during the previous 3 months, being smokers, suffering from cardiometabolic disease, and/or being pregnant. The baseline assessment of the participants of the FIT-AGEING study was conducted during September (2016 and 2017) in the Sport and Health Joint University Institute (iMUDS, Granada, Spain). Oral and written informed consent were provided to all participants accordingly with the Declaration of Helsinki (last revision, 2013). The methodology and procedures of the study were approved by the Human Research Ethics Committee of the Junta de Andalucía (No. 0838-N-2017).

### 2.2. Procedures

#### 2.2.1. Anthropometry and Body Composition

A previously-calibrated Seca scale and stadiometer (model 799, Electronic Column Scale, Hamburg, Germany) was used to assess the weight and height of the participants, respectively. Subsequently, the body mass index (BMI) was determined as weight (kg)/height^2^ (m). These measurements were conducted with light clothing and no shoes. Waist circumference was assessed at the midpoint of the iliac crest and the bottom of the rib cage after the completion of an expiration phase. Body composition (i.e., lean mass, fat mass, and visceral adipose tissue) was determined by dual-energy X-ray absorptiometry (Discovery Wi, Hologic, Inc, Bedford, MA, USA) following the manufacturer’s guidelines. Lean mass index and fat mass index were calculated as lean mass in kg divided by height^2^ in meters, and fat mass in kg divided by height^2^ in meters, respectively.

#### 2.2.2. Basal Metabolic Rate and Basal Nutrients Oxidation

BMR, BCHox, and BFox assessments were performed between 7:00 a.m. and 11:00 a.m. Participants came to the lab in a motorized vehicle avoiding any physical activity after waking up in a fasted state (~8 h). We standardized the evening meal of the previous day: boiled rice plus egg omelet and tomato purée. Participants were instructed to avoid moderate (i.e., 24 h) and vigorous (i.e., 48 h) physical activity before the testing day. Before the beginning of the measurements, the participants had to confirm that they met the aforementioned study conditions. BMR, BFox, and BCHox were estimated by indirect calorimetry using an ergospirometry system based on mixed expired gas (Ultima CardiO2, Medgraphics Corp, St. Paul, MN, USA) and a neoprene face mask without external ventilation. This device measures VO_2_ and VCO_2_ using a breath-by-breath technique for determining the gas exchange.

The indirect calorimetry measurements followed current methodological guidelines [26]. Briefly, all assessments were performed in a quiet room, with a predetermined ambient temperature (22–24 °C) and humidity (35–45%). The participants were instructed to breathe normally, neither talking, fidgeting, nor sleeping during the test, and were positioned on a reclined bed in a supine position covered by a sheet resting for 20 min before the beginning of the register. BMR, BFox, and BCHox were assessed over a 30 min period discarding the first 5 min of each dataset [26]. Flow calibration was performed using a 3-L calibration syringe, and gas analyzers calibration was conducted using 2 standard gas concentrations strictly following the manufacturer’s recommendations.

The most stable 5-min period that met steady-state criteria (i.e., coefficient of variation <10% in VO_2_, CO_2_, and minute ventilation, and coefficient of variation <10% in respiratory exchange ratio (RER)) was considered for further analysis [4,5,27,28]. Participants with an RER lower than 0.7 and/or higher than 1.0 were excluded from the final analysis. BMR, BFox, and BCHox were estimated using the Weir abbreviated equation ($BMR=3.941\times VO_{2}+1.106\times VCO_{2}-2.17*N)$ [29] and the Frayn equation ($BFox=1.67\times VO_{2}+1.67\times VCO_{2}-1.92*N\;and\;BCHox=4.55\times VO_{2}+3.21\times VCO_{2}-2.87*N)$ [30], respectively. We expressed BMR in absolute values (kcal/d) whereas BFox and BCHox were expressed in absolute values (g/min), and as a percentage of the BMR.

#### 2.2.3. Blood Pressure

The updated European Heart Society guidelines for assessing blood pressure were strictly followed [31]. Concretely, an automatic monitor (Omrom^®^ HEM 705 CP, Health-care Co, Kyoto, Japan) was used to measure blood pressure after ~30 min lying on a reclined bed. A total of 3 trials separated by 1 min were performed in the right arm, and the average of systolic and diastolic blood pressure was considered in all cases.

#### 2.2.4. Blood Samples

Blood samples were taken from the antecubital vein just before the BMR and nutrients oxidation test (i.e., between 7:00 a.m. and 11:00 a.m. and after ~8 h fasting). They were stored into EDTA tubes (Vacutainer SST, Becton Dickinson, Plymouth, UK), subsequently centrifuged (i.e., 4000 rpm at 4 °C for 7 min), and finally frozen at −80 °C for further analysis.

Plasma glucose and insulin were determined using standard techniques (AU5800, Beckman Coulter, Brea, CA, USA, and UniCel DxI 800, Beckman Coulter, Brea, CA, USA, respectively). The insulin glucose ratio, the quantitative insulin sensitivity check index (QUICKI = the inverse of the sum of the logarithms of the plasma insulin and plasma glucose) [32], and the homeostatic model assessment of insulin resistance index (HOMA = plasma insulin (UI/mL) × plasma glucose (nmol/L)/22.5)) [33] were subsequently calculated. Total cholesterol, high-density lipoprotein cholesterol (HDL-C), and low-density lipoprotein cholesterol (LDL-C) were assessed using routine procedures (AU5800, Beckman Coulter, Brea, CA, USA). The LDL-H/HDL-H ratio, and the triglycerides/HDL-C ratio were also calculated.

#### 2.2.5. Metabolic Syndrome

Participants were accordingly classified based on (i) their BMI as normal-weight (BMI ranged from 18.5–24.9) or overweight–obese (BMI ≥ 25) and (ii) as metabolically healthy or metabolically unhealthy phenotype [34]. The metabolically unhealthy phenotype was considered when the participants showed at least one of the following criteria: serum triglyceride ≥150 mg/dL, HDL-C < 40 mg/dL for men and 50 mg/dL for women, systolic blood pressure ≥130 mmHg or diastolic blood pressure ≥85 mmHg, or serum glycemia >100 mg/dL [35]. In summary, the metabolically healthy normal-weight phenotype was considered when the participants had a BMI > 18.5 but <25 and did not present any of the above-mentioned risk factors, while the metabolically unhealthy normal-weight phenotype was considered when the participants had a BMI > 18.5 but <25 and showed at least one of the above-mentioned risk factors. Similarly, the metabolically healthy overweight–obese phenotype was considered when the participants had a BMI ≥ 25 and did not present any of the above-mentioned risk factors, while the metabolically unhealthy overweight–obese phenotype was considered when the participants had a BMI ≥ 25 and showed at least one of the above-mentioned risk factors.

#### 2.2.6. Dietary Intake

Dietary intake assessment was performed by 3 non-consecutive 24-h recalls (one weekend day included) conducted by an experienced and qualified nutritionist [24]. The interviews were meal-sequence based and involved a detailed measurement and description of the food ingested. Colored photographs of different portion sizes of foods were provided to help estimate the quantity of meal consumed. Energy consumption and macronutrient intake were determined by using the EvalFINUT^®^ software (Granada, Spain), which includes the USDA (U.S. Department of Agriculture) and BEDCA (“Base de Datos Española de Composición de Alimentos”) databases.

#### 2.2.7. Sedentary Behavior and Physical Activity Levels

Sedentary behavior and physical activity levels were assessed by a wrist-worn accelerometer (ActiGraph GT3X+, Pensacola, FL, USA) during 7 consecutive days (24 h/d). The accelerometers were given to the participants with specific instructions about how to wear it. They were also asked to remove the accelerometer in water-based activities. We selected 100 Hz as the sampling frequency to store raw accelerations. Data were processed by the ActiLife v. 6.13.3 software (ActiGraph, Pensacola, FL, USA) and the GGIR package (v. 1.5-12, https://cran.r-project.org/web/packages/GGIR/) in R (v. 3.1.2, https://www.cran.r-project.org/) following the updated methodological guidelines [36,37]. Processing methods included: (i) a local gravity data auto-calibration, (ii) the determination of the Euclidean Norm Minus One, (iii) the assessment of non-wear time based on the raw acceleration of the 3 axes, (iv) the identification of malfunctioning of the accelerometer based on abnormal accelerations, (v) imputation of non-wear time and abnormal accelerations, (vi) determination of waking and sleeping time by an automatized algorithm guided by the daily reports of the participants, and (vii) the determination of sedentary time, light physical activity time, moderate physical activity time, and vigorous activity time using age-specific cut-points for Euclidean Norm Minus One [38]. We considered time in moderate–vigorous physical activity for further analyses. Participants that did not wear the accelerometers for more than 16 h/d during more than 4 days were excluded for the final analysis.

### 2.3. Statistical Analysis

Descriptive parameters of the participants are expressed as mean and standard deviation, otherwise stated. Visual check of histograms, Shapiro–Wilk test, box plots, and Q–Q plots were performed to check the normal distribution of the study variables. Data presenting non-normal distribution were transformed by taking Napierian logarithm. Unpaired Student *t*-tests were used to test differences between men and women.

We conducted single linear regressions to study the association of BMR, BFox, and BCHox with cardiometabolic risk factors (i.e., blood pressure and glycemic and lipid profile). In addition, multiple linear regression analyses models were built to investigate the above-mentioned associations after controlling for potential confounders (i.e., sex, age, and lean mass). Potential confounders were selected based on statistical procedures (i.e., hierarchical regressions). Sex, age, and lean mass were included as a covariate, while dietary intake and physical-activity-related parameters were excluded from multiple linear regression models.

We used analyses of variance to compare BMR, BFox, and BCHox between metabolically healthy normal-weight, metabolically unhealthy normal-weight, metabolically healthy overweight–obese, and metabolically unhealthy overweight–obese participants. Analyses of covariance were performed to test whether BMR, BFox, and BCHox were different between the above-mentioned phenotypes controlling by sex, age, and lean mass.

The statistical analyses were performed using the Statistical Package for the Social Sciences (SPSS version 24, Inc. Chicago, IL, USA) while the GraphPad Prism 5 (GraphPad Software, San Diego, CA, USA) was used to build the graphical plots. The level of significance was set at *p* ≤ 0.05.

## 3. Results

Table 1 shows the descriptive characteristics of the participants by sex.

We observed positive associations of BMR with plasma insulin, HOMA, and LDL-C/HDL-C (all *p* < 0.05; Table 2) which were attenuated or disappeared after controlling by sex, age, and/or lean mass (Table 2). There were positive associations between BFox (expressed in absolute terms and as a % of BMR) and QUICKI (*p* < 0.015; Table 2), while negative associations were noted between BFox and plasma insulin and HOMA (all *p* < 0.015; Table 2). These associations persisted in almost all cases when controlling by sex, age, and/or lean mass (Table 2). No further relationships were found when BMR and BFox were associated with other cardiometabolic risk factors (Table 2).

There was a significant negative association between BCHox (expressed in absolute terms and as a % of BMR) with QUICKI (all *p* < 0.01; Table 3), whereas significant positive relationships were obtained when BCHox was associated with plasma insulin and HOMA (all *p* < 0.01; Table 3). These associations persisted in almost all cases after adjusting by sex, age, and/or lean mass (Table 3). No further relationships were found when BCHox was associated with other cardiometabolic risk factors (Table 3).

**Figure 1:**
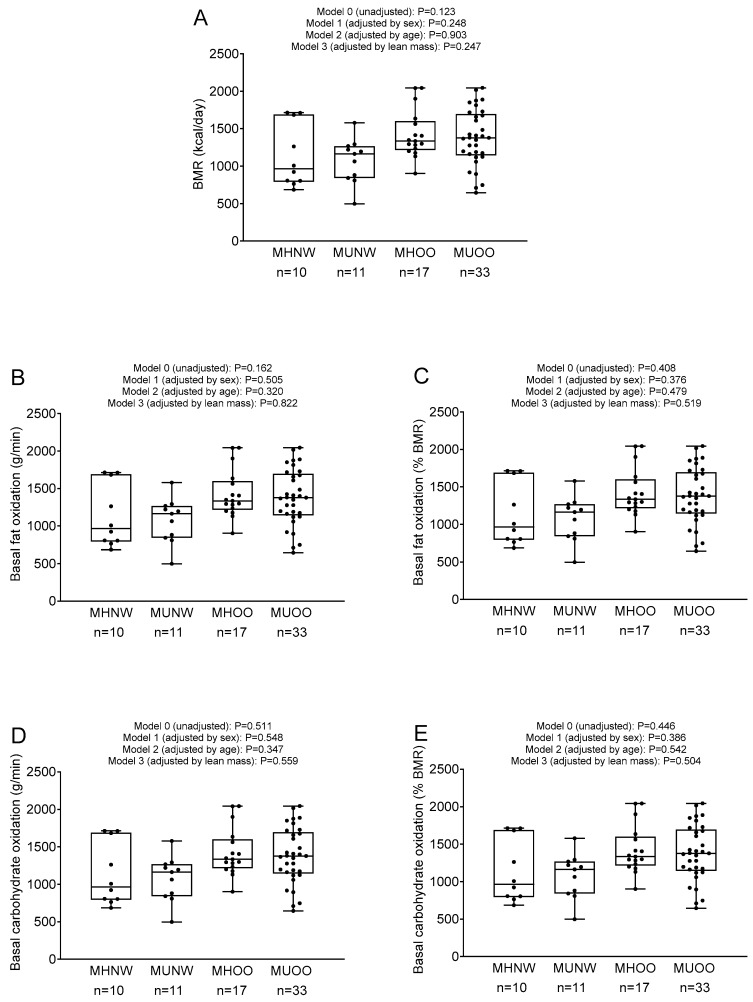
(**A**) Basal metabolic rate (BMR), (**B**,**C**) basal fat oxidation, and (**D**,**E**) basal carbohydrate oxidation in metabolically healthy normal-weight (MHNW), metabolically unhealthy normal–weight (MUNW), metabolically healthy overweight–obese (MHOO), and metabolically unhealthy overweight–obese (MHOO) participants. *P* from analysis of variance (Model 0) and analysis of covariance (Model 1: adjusting by sex; Model 2: adjusting by age; Model 3: adjusting by lean mass).

BMR, BFox, and BCHox were similar in metabolically healthy normal-weight, metabolically unhealthy normal-weight, metabolically healthy overweight–obese and metabolically unhealthy overweight–obese individuals independently of sex, age, and lean mass (all *p* > 0.12; Figure 1).

## 4. Discussion

The main findings of the present study suggest that BFox is associated with better insulin sensitivity in sedentary middle-aged adults, whereas BCHox is positively related to insulin resistance. However, no further associations were noted of BFox and BCHox with other cardiometabolic risk factors. Moreover, no association was found between BMR neither with cardiometabolic risk factors nor insulin sensitivity in our study cohort.

We showed that BMR was not associated with glucose homeostasis. Our results did not concur with those obtained by other studies that demonstrated that BMR was positively associated with plasma glucose, glucose tolerance, and insulin sensitivity in adults [15,16,39]. Patients with diabetes type 2 show an increased BMR probably due to the higher gluconeogenetic activity [40]. This BMR increment may be due to increased gluconeogenesis and hepatic glucose output, high protein metabolism, high oxidation levels for carbohydrate metabolism, activation of the sympathetic nervous system, and altered metabolic activities [17,39]. Moreover, there is a subset of obese individuals without classical cardiometabolic disturbances (i.e., metabolically healthy obesity phenotype) [41,42]. Therefore, although these subjects present obesity, it would be plausible that they show better levels of BMR and nutrients oxidation compared to their metabolically unhealthy counterparts. Unexpectedly, no significant differences in BMR and nutrients oxidation were noted between metabolically healthy and metabolically unhealthy participants independently of their BMI status. These controversial results could be explained because the above-mentioned mechanisms may not be applicable to relatively-healthy individuals like our participants since it is previously shown that pathological status (i.e., diabetes type II) have an impaired metabolic status, energy homeostasis, and insulin clearance rate [43]. A better understanding of the mechanisms underlying the relationship between BMR and glucose homeostasis parameters in healthy adults is, therefore, desirable.

Metabolic inflexibility is characterized by impaired fat oxidation in basal conditions [3]. In obesity, there is compromised metabolic flexibility due to the deteriorated insulin-mediated substrate switching [10]. In addition, there is impaired fatty acid oxidation in myocardial ischemia, ventricular hypertrophy, systemic hypertension patients, obesity, metabolic syndrome, and diabetes type II patients [10]. Therefore, it seems plausible that BFox and BCHox could be associated with the cardiometabolic risk score and glucose homeostasis parameters. Fatty acids are the main energy substrate in the heart [10]. The failing capacity to use fatty acids and an increased dependence on glucose oxidation could cause cardiac metabolic inflexibility [10]. This cardiac metabolic inflexibility affects ATP production and cardiac contractility, triggers mitochondrial dysfunction which affects myocardial metabolism and deteriorates cardiac performance consequently, increasing cardiovascular risk [44]. Furthermore, it has been proposed that a decreased BFox and an increased BCHox may precede insulin resistance [10], being metabolic inflexibility at the core of the pathophysiology of insulin resistance [10]. Impaired fatty acid oxidation could induce lipid metabolites accumulation (e.g., diacylglycerol and ceramides), which may increase serine phosphorylation of the insulin receptor, decrease activation of Akt/PKB, and alter mitochondrial energetics leading to an impaired insulin signaling [10]. Therefore, although our participants were healthy (i.e., free of cardiometabolic diseases), our results support the notion that BFox is a good marker of insulin sensitivity in healthy adults.

The findings of the current study should be cautiously interpreted since it presents some limitations. No causal interpretation should be done since our study has a cross-sectional design. Our study presents a relatively low sample size. Although accelerometry is considered the gold standard method to assess physical activity, it presents some limitations (e.g., arm movements during activities of daily living would increase the total daily number of counts, which can be interpreted as physical activity). The participants were sedentary adults (aged from 45 to 65 years old), and whether these findings apply to younger, older, or physically active individuals remains unknown. In addition, these results should not be extended to patients with cardiometabolic diseases (i.e., diabetes mellitus type II or heart disease). Finally, the assessment of blood parameters in the late morning (i.e., 11:00 a.m.) in some participants should be considered as a potential limitation.

In summary, our study findings support that greater BFox and lower BCHox are related to improved insulin sensitivity, whereas BMR seems to be not associated with neither cardiometabolic risk nor insulin sensitivity in sedentary middle-aged adults. These results have an important clinical impact since improving nutrients oxidation in fasting conditions through different strategies (i.e., physical exercise or dietary interventions) could provide cardiometabolic protection to sedentary but healthy middle-aged adults. Further intervention studies are necessary to properly understand the physiological mechanism implied in this relationship.

## Figures and Tables

**Table 1:** Descriptive characteristics of the study participants.

	All (*n* = 71)		Men (*n* = 34)		Women (*n* = 37)	
Age (years)	53.4	(4.9)	54.2	(5.2)	52.7	(4.6)
Anthropometry and body composition	(*n* = 71)		(*n* = 34)		(*n* = 37)	
Body mass index (kg/m^2^)	26.82	(3.79)	28.50	(3.51)	25.29	(3.40) *
Waist circumference (cm)	95.29	(11.89)	103.13	(8.45)	88.09	(9.91) *
Lean mass index (kg/m^2^)	15.29	(2.86)	17.49	(2.06)	13.26	(1.78) *
Fat mass index (kg/m^2^)	10.74	(3.10)	10.15	(3.20)	11.28	(2.95) *
Visceral adipose tissue mass (g)	788.9	(391.8)	986.4	(388.8)	607.4	(298.7) *
Basal metabolic rate	(*n* = 68)		(*n* = 34)		(*n* = 34)	
BMR (kcal/d)	1323	(376)	1548	(305)	1098	(301) *
BMR (kcal/kg_leanmass_/d)						
Nutrients oxidation	(*n* = 57)		(*n* = 28)		(*n* = 29)	
BFox (g/min)	0.116	(0.098)	0.135	(0.117)	0.098	(0.074) *
BFox (% BMR)	48.32	(35.72)	47.90	(38.13)	48.73	(33.91)
BCHox (g/min)	0.112	(0.096)	0.137	(0.115)	0.088	(0.069) *
BCHox (% BMR)	48.06	(34.34)	50.00	(37.33)	46.28	(31.77)
Blood pressure	(*n* = 67)		(*n* = 31)		(*n* = 36)	
Systolic (mm Hg)	127.09	(15.78)	134.26	(13.84)	120.92	(14.85) *
Diastolic (mm Hg)	81.12	(11.72)	85.16	(10.87)	77.64	(11.44) *
Mean (mm Hg)	104.10	(13.15)	109.71	(11.70)	99.28	(12.52) *
Glycemic profile	(*n* = 70)		(*n* = 33)		(*n* = 37)	
Plasma glucose (mg/dL)	93.56	(11.36)	95.00	(13.60)	92.27	(8.90)
Plasma insulin (UI/mL)	8.08	(5.68)	8.94	(6.75)	7.32	(4.48)
QUICKI	0.362	(0.036)	0.357	(0.039)	0.365	(0.033)
HOMA	1.933	(1.668)	2.209	(2.107)	1.686	(1.120) *
Lipid profile	(*n* = 70)		(*n* = 33)		(*n* = 37)	
Total cholesterol (mg/dL)	206.14	(32.17)	200.67	(32.29)	211.03	(31.70)
HDL-C (mg/dL)	58.71	(12.28)	55.33	(12.86)	61.73	(11.06) *
LDL-C (mg/dL)	131.39	(64.57)	138.79	(78.00)	124.78	(49.88)
Triglycerides (mg/dL)	126.23	(27.07)	125.06	(27.93)	127.27	(26.63)
LDL-C/HDL-C	2.234	(0.632)	2.358	(0.667)	2.123	(0.585) *
Dietary intake	(*n* = 70)		(*n* = 34)		(*n* = 36)	
Energy (kcal/d)	2140	(699)	2396	(841)	1901	(417) *
Fat (g/d)	87.8	(24.9)	98.2	(23.8)	78.0	(22.0) *
Carbohydrate (g/d)	227.9	(113.5)	255.9	(145.6)	201.4	(62.6) *
Protein (g/d)	88.8	(38.2)	94.2	(39.1)	83.6	(37.2) *
Sedentary behaviour and physical activity	(*n* = 70)		(*n* = 34)		(*n* = 36)	
Sedentary time (min/d)	746.9	(84.3)	770.0	(80.3)	725.1	(83.3)
Time in MVPA (min/d)	95.9	(35.6)	96.6	(35.5)	95.2	(36.2)

Data are shown as means (standard deviation). * Significant differences between sexes (Student’s unpaired *t*-test; *p* < 0.05). Abbreviations: BMR; basal metabolic rate, BFox; basal fat oxidation, BCHox; basal carbohydrate oxidation QUICKI; quantitative insulin sensitivity check index, HOMA; homeostasis model assessment index, HDL-C; high-density lipoprotein cholesterol, LDL-C; low-density lipoprotein cholesterol, MVPA: moderate–vigorous physical activity.

**Table 2:** Association of basal metabolic rate and basal fat oxidation with cardiometabolic risk factors.

	Model 0				Model 1				Model 2				Model 3			
	F_1_,_68_	*p* value	R^2^	β	F_1_,_67_	*p* value	R^2^	β	F_1_,_67_	*p* value	R^2^	β	F_1_,_67_	*p* value	R^2^	β
**Basal Metabolic Rate (kcal/d)**																
Systolic blood pressure (mm Hg)	1.243	0.269	0.004	0.137	8.377	0.145	0.183	−0.210	0.753	0.231	−0.008	0.155	6.062	0.523	0.133	−0.086
Diastolic blood pressure (mm Hg)	1.359	0.248	0.005	0.143	3.933	0.530	0.082	−0.095	0.710	0.238	−0.009	0.153	3.731	0.834	0.076	−0.029
Mean blood pressure (mm Hg)	1.415	0.239	0.006	0.146	6.844	0.250	0.150	−0.169	0.797	0.213	−0.006	0.161	5.498	0.634	0.120	−0.064
Plasma glucose (mg/dL)	1.017	0.317	0.001	0.121	0.618	0.626	0.018	0.076	0.134	0.291	−0.013	0.134	0.504	0.362	−0.015	0.126
Plasma insulin (UI/mL)	**4.101**	**0.047**	**0.043**	**0.238**	2.022	0.112	0.029	0.243	5.472	0.181	0.115	0.159	**3.390**	**0.013**	**0.065**	**0.339**
QUICKI	3.652	0.060	0.037	−2.226	1.854	0.096	0.024	−0.256	4.612	0.203	0.095	−0.153	**2.822**	**0.021**	**0.078**	**−0.314**
HOMA	**5.528**	**0.022**	**0.062**	**0.274**	**3.091**	**0.021**	**0.084**	**0.350**	4.836	0.078	0.100	0.212	**4.048**	**0.006**	**0.081**	**0.371**
Total cholesterol (mg/dL)	0.086	0.771	−0.013	−0.035	1.145	0.494	0.004	0.105	2.533	0.380	0.043	−0.108	3.705	0.315	0.073	0.133
HDL-C (mg/dL)	3.361	0.071	0.033	−0.217	2.655	0.555	0.046	−0.089	**3.003**	**0.030**	**0.055**	**−0.269**	4.290	0.551	0.087	−0.078
LDL-C (mg/dL)	1.143	0.289	0.002	0.129	1.402	0.106	0.012	0.250	2.175	0.569	0.061	0.070	2.844	0.063	0.051	0.262
Triglycerides (mg/dL)	0.063	0.802	−0.014	−0.030	0.947	0.304	−0.002	−0.159	0.379	0.984	−0.018	−0.002	0.361	0.547	−0.019	−0.084
LDL-C/HDL-C	**4.378**	**0.040**	**0.047**	**0.246**	2.229	0.165	0.034	0.212	2.207	0.059	0.034	0.236	2.254	0.108	0.035	0.219
	F_1,56_	*p* value	R^2^	β	F_1_,_55_	*p* value	R^2^	β	F_1_,_55_	*p* value	R^2^	β	F_1_,_55_	*p* value	R^2^	β
**Basal Fat Oxidation (g/min)**																
Systolic blood pressure (mm Hg)	2.781	0.100	0.026	0.203	7.484	0.401	0.164	0.099	1.467	0.094	0.014	0.224	5.937	0.657	0.156	0.056
Diastolic blood pressure (mm Hg)	2.594	0.112	0.024	0.196	4.254	0.327	0.090	0.120	1.519	0.086	0.015	0.229	3.927	0.531	0.081	0.081
Mean blood pressure (mm Hg)	2.964	0.090	0.029	0.209	6.585	0.343	0.145	0.113	1.626	0.077	0.019	0.236	5.544	0.582	0.148	0.070
Plasma glucose (mg/dL)	1.102	0.297	0.001	−0.126	1.427	0.180	0.012	−0.167	0.669	0.253	−0.010	−0.149	1.102	0.297	0.001	−0.126
Plasma insulin (UI/mL)	**6.559**	**0.013**	**0.075**	**−0.297**	**5.392**	**0.003**	**0.113**	**−0.355**	5.992	0.102	0.126	−0.200	**3.420**	**0.012**	**0.066**	**−0.326**
QUICKI	**6.379**	**0.014**	**0.072**	**0.293**	**4.645**	**0.005**	**0.096**	**0.342**	5.294	0.094	0.111	0.206	**3.419**	**0.012**	**0.066**	**0.328**
HOMA	**8.211**	**0.006**	**0.108**	**−0.328**	**5.507**	**0.002**	**0.116**	**−0.376**	**5.546**	**0.038**	**0.116**	**−0257**	**4.525**	**0.004**	**0.093**	**−0.373**
Total cholesterol (mg/dL)	2.321	0.132	0.019	−0.182	1.667	0.226	0.019	−0.150	2.509	0.395	0.042	−0.108	3.366	0.526	0.064	−0.080
HDL-C (mg/dL)	0.965	0.329	0.014	−0.118	2.579	0.648	0.044	−0.056	0.734	0.513	−0.008	−0.085	4.092	0.931	0.082	0.011
LDL-C (mg/dL)	1.611	0.209	0.009	−0.152	0.794	0.229	−0.006	−0.151	2.196	0.546	0.034	−0.077	1.203	0.414	0.006	−0.107
Triglycerides (mg/dL)	0.827	0.366	−0.003	0.110	0.652	0.486	−0.010	0.088	0.582	0.528	−0.012	0.082	0.444	0.469	−0.016	0.096
LDL-C/HDL-C	0.033	0.856	−0.014	−0.022	1.394	0.553	0.011	−0.074	0.348	0.903	−0.019	0.016	1.191	0.310	0.006	−0.099
**Basal Fat Oxidation (% Basal Metabolic Rate)**																
Systolic blood pressure (mm Hg)	0.853	0.359	−0.002	0.114	7.848	0.257	0.172	0.128	0.444	0.362	−0.017	0.127	6.046	0.538	0.133	0.071
Diastolic blood pressure (mm Hg)	0.450	0.505	−0.008	0.083	4.066	0.428	0.085	0.094	0.290	0.450	−0.022	0.105	3.797	0.687	0.078	0.048
Mean blood pressure (mm Hg)	0.728	0.397	−0.004	0.105	6.692	0.300	0.147	0.119	0.402	0.377	−0.018	0.123	5.544	0.582	0.121	0.064
Plasma glucose (mg/dL)	1.426	0.237	0.006	−0.143	1.178	0.251	0.005	−0.139	0.937	0.177	−0.002	−0.183	0.862	0.216	−0.004	−0.152
Plasma insulin (UI/mL)	**8.336**	**0.005**	**0.096**	**−0.330**	**4.853**	**0.006**	**0.100**	**−0.326**	**6.263**	0.077	0.132	−0.224	**4.112**	**0.006**	**0.083**	**−0.329**
QUICKI	**6.853**	**0.011**	**0.078**	**0.303**	**3.781**	**0.012**	**0.075**	**0.299**	5.137	0.112	0.107	0.204	**3.376**	**0.012**	**0.064**	**0.303**
HOMA	**10.262**	**0.002**	**0.118**	**−0.362**	**5.354**	**0.002**	**0.112**	**−0.359**	**6.041**	**0.023**	**0.127**	**−0.291**	**5.066**	**0.002**	**0.105**	**−0.364**
Total cholesterol (mg/dL)	1.320	0.255	0.005	−0.138	1.647	0.232	0.018	−0.144	2.165	0.768	0.033	−0.039	3.573	0.379	0.069	−0.104
HDL-C (mg/dL)	0.033	0.855	−0.014	−0.022	2.504	0.791	0.042	−0.031	0.557	0.773	−0.013	0.039	4.103	0.873	0.083	0.019
LDL-C (mg/dL)	1.905	0.172	0.013	−0.165	1.106	0.171	0.001	−0.167	2.177	0.566	0.033	−0.076	1.633	0.223	0.018	−0.148
Triglycerides (mg/dL)	0.413	0.523	−0.009	0.078	0.633	0.503	−0.011	0.082	0.421	0.774	−0.017	0.039	0.340	0.570	−0.019	0.070
LDL-C/HDL-C	0.531	0.469	−0.007	−0.088	1.450	0.498	0.013	−0.082	0.423	0.687	−0.017	−0.055	1.315	0.368	0.009	−0.109

F, β (standardized regression coefficient), R^2^ (standardized) and *p* from simple and multiple linear regression analyses. Model 0: simple regression analysis; Model 1: regression analysis including sex as a covariate; Model 2: regression analysis including age as a covariate; Model 3: regression analysis including lean mass as a covariate. Statistically significant associations are presented in bold. Abbreviations: QUICKI; quantitative insulin sensitivity check index; HOMA; homeostasis model assessment index, HDL-C; high-density lipoprotein cholesterol, LDL-C; low-density lipoprotein cholesterol.

**Table 3:** Association of basal carbohydrate oxidation with cardiometabolic risk factors.

	Model 0				Model 1				Model 2				Model 3			
	F_1,56_	*p* value	R^2^	β	F_1_,_55_	*p* value	R^2^	β	F_1_,_55_	*p* value	R^2^	β	F_1_,_55_	*p* value	R^2^	β
**Basal Carbohydrate Oxidation (g/min)**																
Systolic blood pressure (mm Hg)	0.046	0.832	−0.015	−0.026	8.109	0.192	0.177	−0.153	0.031	0.889	−0.030	−0.019	6.030	0.552	0.132	−0.069
Diastolic blood pressure (mm Hg)	0.136	0.713	−0.013	−0.046	4.490	0.242	0.096	−0.144	0.087	0.679	−0.028	−0.057	3.966	0.497	0.110	−0.081
Mean blood pressure (mm Hg)	0.086	0.771	−0.014	−0.036	7.088	0.190	0.156	−0.156	0.042	0.788	−0.030	−0.037	5.626	0.506	0.123	−0.078
Plasma glucose (mg/dL)	1.159	0.285	0.002	0.129	0.844	0.435	0.025	0.104	0.721	0.235	−0.008	0.157	0.618	0.305	−0.011	0.126
Plasma insulin (UI/mL)	**7.196**	**0.009**	**0.082**	**0.309**	**3.690**	**0.018**	**0.072**	**0.292**	6.105	0.091	0.129	0.209	**3.828**	**0.008**	**0.076**	**0.318**
QUICKI	**6.991**	**0.010**	**0.080**	**−0.305**	**3.472**	**0.016**	**0.067**	**−0.298**	5.416	0.083	0.113	−0.216	**3.631**	**0.009**	**0.071**	**−0.312**
HOMA	**11.156**	**0.001**	**0.128**	**0.375**	**5.501**	**0.002**	**0.115**	**0.378**	**6.670**	**0.013**	**0.141**	**0.309**	**5.698**	**0.001**	**0.120**	**0.382**
Total cholesterol (mg/dL)	0.639	0.427	−0.005	0.096	1.686	0.221	0.019	0.153	2.118	0.980	0.031	0.003	3.793	0.280	0.075	0.127
HDL-C (mg/dL)	0.049	0.826	−0.014	−0.027	2.555	0.685	0.043	0.050	0.738	0.510	−0.008	−0.087	4.090	0.959	0.082	0.006
LDL-C (mg/dL)	1.767	0.188	0.011	0.011	1.151	0.144	0.004	0.185	2.206	0.536	0.034	0.080	1.979	0.144	0.028	0.176
Triglycerides (mg/dL)	0.516	0.475	−0.007	−0.087	0.921	0.316	−0.002	−0.127	0.465	0.682	−0.016	−0.054	0.481	0.439	−0.015	−0.095
LDL-C/HDL-C	1.214	0.274	0.003	0.132	1.464	0.486	0.013	0.087	1.384	0.332	0.011	0.118	1.384	0.332	0.011	0.118
**Basal Carbohydrate Oxidation (% Basal Metabolic Rate)**																
Systolic blood pressure (mm Hg)	0.046	0.832	−0.015	−0.026	8.109	0.192	0.177	−0.153	0.031	0.889	−0.030	−0.019	6.030	0.552	0.132	−0.069
Diastolic blood pressure (mm Hg)	0.136	0.713	−0.013	−0.046	4.490	0.242	0.096	−0.144	0.087	0.679	−0.028	−0.057	3.966	0.497	0.082	−0.081
Mean blood pressure (mm Hg)	0.086	0.771	−0.014	−0.036	7.088	0.190	0.156	−0.156	0.042	0.788	−0.030	−0.037	5.626	0.506	0.123	−0.078
Plasma glucose (mg/dL)	1.159	0.285	0.002	0.129	0.844	0.411	−0.005	0.104	0.721	0.235	−0.008	0.157	0.618	0.305	−0.011	0.126
Plasma insulin (UI/mL)	**7.196**	**0.009**	**0.082**	**0.309**	**3.690**	**0.018**	**0.072**	**0.292**	6.105	0.091	0.129	0.209	**3.828**	**0.008**	**0.076**	**0.318**
QUICKI	**6.991**	**0.010**	**0.080**	**−0.305**	**4.472**	**0.016**	**0.067**	**−0.298**	5.416	0.083	0.113	−0.216	**3.631**	**0.009**	**0.071**	**−0.312**
HOMA	**11.156**	**0.001**	**0.128**	**0.375**	**5.501**	**0.002**	**0.115**	**0.378**	**6.670**	**0.013**	**0.141**	**0.309**	**5.698**	**0.001**	**0.120**	**0.382**
Total cholesterol (mg/dL)	0.639	0.427	−0.005	0.096	1.686	0.221	0.019	0.153	2.118	0.980	0.031	0.003	3.793	0.280	0.075	0.127
HDL-C (mg/dL)	0.049	0.826	−0.014	−0.027	2.555	0.685	0.043	0.050	0.738	0.510	−0.008	−0.087	4.090	0.959	0.082	0.006
LDL-C (mg/dL)	1.767	0.188	0.011	0.159	1.151	0.144	0.004	0.185	2.206	0.536	0.034	0.080	1.979	0.144	0.028	0.176
Triglycerides (mg/dL)	0.516	0.475	−0.007	−0.087	0.921	0.316	−0.002	−0.127	0.465	0.682	−0.016	−0.054	0.481	0.439	−0.015	−0.095
LDL-C/HDL-C	1.214	0.274	0.003	0.132	1.464	0.486	0.013	0.087	0.698	0.403	−0.009	0.113	1.384	0.332	0.011	0.118

F, β (standardized regression coefficient), R^2^ (standardized) and *p* from simple and multiple linear regression analyses. Model 0: simple regression analysis; Model 1: regression analysis including sex as a covariate; Model 2: regression analysis including age as a covariate; Model 3: regression analysis including lean mass as a covariate. Statistically significant associations are presented in bold. Abbreviations: QUICKI; quantitative insulin sensitivity check index; HOMA; homeostasis model assessment index, HDL-C; high-density lipoprotein cholesterol, LDL-C; low-density lipoprotein cholesterol.
